# Effect of recurrent invasive pneumococcal disease on serum anti-pneumolysin IgG titres in HIV infected adults

**DOI:** 10.1016/j.vaccine.2009.04.026

**Published:** 2009-06-12

**Authors:** Onajite M. Etuwewe, Natalie Swann, Susan Hollingshead, Helen Tolmie, Ed E. Zijlstra, Brian Faragher, Neil French, Stephen B. Gordon

**Affiliations:** a Alder Hey Children's NHS Foundation Trust, Liverpool, UK; b Liverpool School of Tropical Medicine, Liverpool, UK; c University of Alabama, Birmingham, USA; d Department of Medicine, College of Medicine, Blantyre, Malawi; e Karonga Prevention Study, Chilumba, Malawi

**Keywords:** HIV infection, Pneumococcal bacteraemia, Pneumolysin

## Abstract

We measured serum anti-pneumolysin IgG concentrations in a prospective cohort of 34 HIV infected adults who developed recurrent pneumococcal bacteraemia, and compared baseline levels with HIV positive and HIV negative control subjects that remained free of pneumococcal disease. Anti-pneumolysin concentrations in HIV positive cases and controls were higher compared to HIV negative controls. There was no significant difference in levels between HIV positive subjects who did and did not subsequently develop pneumococcal bacteraemia (geometric means 849.1 U/ml vs. 564.6 U/ml, *p* = 0.059). Anti-pneumolysin IgG titres before, and after the recurrent episode of pneumococcal bacteraemia did not differ significantly (*p* = 0.95). High levels of anti-pneumolysin IgG do not predict protection from invasive pneumococcal disease or indicate that an effective immune response has occurred in HIV infected patients.

## 1 Introduction

HIV infected individuals have higher rates of severe, recurrent pneumococcal disease compared to HIV uninfected adults [1], contributing to the morbidity and mortality with advanced HIV disease. A recently performed clinical trial has recently shown that a seven-valent pneumococcal conjugate vaccine is effective for the secondary prevention of disease in HIV infected adults caused by vaccine serotypes (N. French in preparation, presented at ISPPD6). However, because of the limited serotype coverage of currently available pneumococcal conjugate polysaccharide vaccines, an improved vaccine strategy to provide broad protection against pneumococcal infection is sought. Protein and protein–polysaccharide components are amongst promising antigens for new pneumococcal vaccines.

*Streptococcus pneumoniae* expresses a potent toxin, pneumolysin. This cytosolic protein is released during bacterial growth and lysis [2,3]. As pneumolysin is common to virtually all *S. pneumoniae* isolates, toxoided derivatives of pneumolysin are main candidates for a protein-based or protein-polysaccharide conjugate pneumococcal vaccine for humans [4].

Antibodies to pneumolysin are produced early in life in response to pneumococcal carriage and infection [5,6]. The protective effect of vaccination with pneumolysin against pneumococcal infection has been demonstrated in animal studies [6]. In humans, colonized subjects and patients with nonbacteraemic pneumococcal pneumonia had higher levels of IgG to pneumolysin compared to patients with bacteraemic pneumococcal pneumonia suggesting that early in infection, anti-pneumolysin protects the host against bacteraemic pneumococcal disease and disease severity [6]. Similarly, in a group of HIV infected patients, a lower baseline level of antibody to pneumolysin was associated with a higher incidence of pneumococcal bacteraemia [7].

The antibody response to pneumolysin in recurrent pneumococcal bacteraemia in HIV infected adults has not been described previously to our knowledge. We measured serum anti-pneumolysin IgG titres in HIV infected Malawian adults followed prospectively for recurrence of pneumococcal bacteraemia. Comparison was made with HIV negative and HIV positive subjects remaining free of invasive pneumococcal disease. The aim of the study was to contribute to experimental information about the utility of pneumolysin as a vaccine for the prevention of invasive pneumococcal disease in HIV infected individuals.

## 2 Methods

### 2.1 Subjects and samples

Thirty-four HIV infected patients with a microbiologically confirmed episode of invasive pneumococcal disease were recruited and followed until they developed a recurrent episode of pneumococcal bacteraemia. These individuals were participants in a randomised controlled trial of seven-valent pneumococcal protein conjugate vaccine, in Blantyre, Malawi. Serum samples were taken at the time of recruitment and then every three months. All 34 cases had at least one further episode of pneumococcal bacteraemia during the study. Recruitment started in February 2003 and finished at the end of May 2007.

HIV infected subjects without a history of pneumococcal disease and HIV-uninfected subjects were identified in a separate cohort of individuals participating in parallel studies of pneumococcal immunity in Blantyre. This was an open cohort recruited during the same interval as the cases and followed up using the same clinic protocols as the vaccine trial participants. Each of the 34 cases was matched individually by age and sex with 1 HIV positive individual and 1 HIV negative individual. If there were a number of potential matches, the individual with a CD4 cell count closest to the case was used as the control. No episode of invasive pneumococcal disease was diagnosed in these individuals up to the end of the study.

Samples were collected at the Queen Elizabeth Central Hospital, Blantyre, Malawi. The serum sample selected for analysis as baseline for the cases was the stored sample that immediately pre-dated the recurrent pneumococcal event. Sera were stored at −80 °C and shipped frozen to the Liverpool School of Tropical Medicine for ELISA analysis. All procedures were conducted with approval from the College of Medicine, Research Ethics Committee and with signed informed consent from each participant.

### 2.2 Measurement of serum anti-pneumolysin IgG

ELISA plates (NUNC, Wiesbaden, Germany) were coated overnight at 4 °C in phosphate buffered saline (PBS) with pneumolysin supplied by University of Alabama, Birmingham, USA, at a concentration of 0.94 mg/ml. All assays included control wells coated with a bovine serum albumin (BSA) to verify the specificity of the assays for the coating antigen. Plates were washed with PBS containing 0.05% Tween 20 (ELISA wash buffer). Plates were always washed three times. The plates were blocked with 0.1% BSA for one hour at room temperature followed by overnight incubation at 4 °C with the subject's sera in serial three-fold dilutions (1:200–1:145800). Following washing with ELISA wash buffer, the plates were incubated with biotin conjugated goat anti-human immunoglobulin (IgG) serum (Oxford Biotechnology, UK) for two hours at room temperature, washed and then incubated with streptavidin–alkaline phosphatase (Oxford Biotechnology, UK) for one hour at room temperature. Incubation at room temperature occurred with the plates rocking on a horizontal shaker. After a final wash, the plates were developed with p-nitrophenyl phosphate (Sigma, St. Louis, MO, USA). Absorbance was read at 405 nm on an ELISA reader (Opys MR, Dynex Technologies) with data analysis software, Revelation Quicklink version 4.25. Assays were standardized using a pooled human serum sample with a known titre of pneumolysin-specific IgG. The coefficient of variation for each assay was <15%.

### 2.3 HIV test

Serum samples from recruits were tested for HIV by twin ELISA (Abbott Murex HIV-1.2.2 kit and Vironostika HIV Uni-Form II plus O kit, bioMerieux). Discordant samples were repeated and if not resolved were subjected to two rapid tests (Determine, Abbott laboratories, and Uni-Gold, Trinity Biotech, Ireland).

### 2.4 CD4+ T-cell counts

CD4+ T-cell counts were measured using BD FACScount (Becton Dickinson) as per manufacturer's instructions.

### 2.5 Data analysis

SPSS software (version 14.0, SPSS) was used for analysis of the data. Antibody concentrations and CD4 counts were converted to natural logarithms for analysis to obtain approximate normal distributions, so the results for both measures are reported using geometric means. Group comparisons were by one way analysis of variance (ANOVA) followed where appropriate by post hoc Tukey multiple comparison tests. Comparison of pre- and post-bacteraemia antibody concentrations for the recurrent bacteraemia group was by paired-samples Student *t*-tests. Differences between groups were considered significant at the conventional 5% level (*p* ≤ 0.05).

## 3 Results

Thirty-four HIV positive trial participants experienced a recurrent pneumococcal bacteraemia event. In five cases post-disease sera were unavailable for analysis. Demographic and clinical details for the subjects are summarized in Table 1. There were no significant differences between the groups with respect to age and sex distribution, but as expected the HIV infected groups had significantly lower CD4+ cell counts than did the HIV negative subjects.

### 3.1 Comparison of baseline anti-pneumolysin IgG titres between cases and controls

**Fig. 1:**
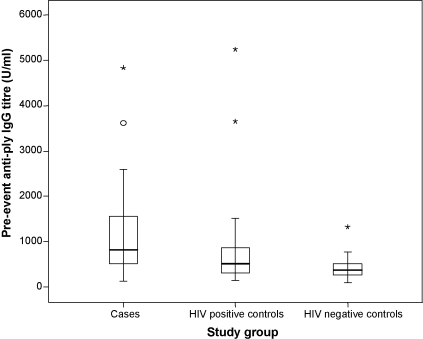
Baseline serum IgG antibodies to pneumolysin in HIV positive cases with recurrent pneumococcal bacteraemia (*n* = 34) matched HIV positive controls (*n* = 34) and matched HIV negative controls (*n* = 34) displayed as box-and-whisker plots. The circle represents an outlier (value between 1.5 and 3 interquartile ranges from the end of the box), asterisks are extreme outliers (>3 interquartile range from box). HIV positive cases and HIV positive controls had significantly higher IgG titres than HIV negative controls. There was no significant difference in levels between cases and HIV positive controls (*p* = 0.059).

HIV positive cases had significantly higher anti-pneumolysin IgG titres than HIV negative subjects (*p* < 0.001). HIV positive controls also had significantly higher anti-pneumolysin IgG titres than the HIV negative controls (*p* = 0.028). HIV positive cases that had recurrence of bacteraemia had a tendency to higher anti-pneumolysin IgG titres compared to HIV positive control subjects (Fig. 1), but this was not statistically significant (*p* = 0.059).

### 3.2 Change in anti-pneumolysin IgG titre in cases after recurrence of pneumococcal bacteraemia

**Fig. 2:**
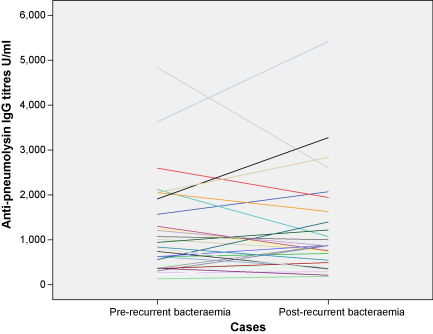
Profile plot of change in specific IgG titres against pneumolysin before (baseline) and after the recurrent pneumococcal bacteraemia episode in the 29 cases with paired titres.

Case sera were collected a median time of 40 days before the recurrent pneumococcal disease episode (range, 0–334 days), for baseline anti-pneumolysin IgG measurement. Sera after the recurrent bacteraemia episode, were collected at a median time of 39 days (range, 13–177 days) post-disease. There was no significant change in anti-pneumolysin IgG titres between baseline level and post-bacteraemia episode (*p* = 0.95) (Fig. 2).

### 3.3 Relationship between CD4+ level and anti-pneumolysin IgG titre

There was no significant difference in CD4 + T-cell counts between HIV positive cases and HIV positive controls (geometric mean 132.9/μl vs. 178.2/μl, *p* = 0.29). A weak but statistically significant negative correlation was found between CD4+ T-cell number and baseline anti-pneumolysin IgG titres in cases and HIV positive controls (*r* = 0.26, *p* = 0.03). There was no significant correlation between CD4+ cell count and change in anti-pneumolysin titre in cases after the bacteraemia episode.

## 4 Discussion

In this study, HIV positive subjects in Malawi had higher anti-pneumolysin titres compared to HIV negative controls. Baseline anti-pneumolysin titres did not differ significantly between HIV positive cases at recruitment after a confirmed pneumococcal bacteraemia episode, prior to recurrence, and HIV positive controls without observed episode of invasive pneumococcal disease. The anti-pneumolysin titres after recurrence of pneumococcal bacteraemia in the cases were not significantly different from baseline values. There was no significant correlation between CD4+ T-cell count and change in anti-pneumolysin titre in cases after the recurrent bacteraemia episode.

Previous studies in different populations with HIV infection in the United States have also reported no significant difference in pre-disease anti-pneumolysin titres between HIV positive cases suffering pneumococcal bacteraemia and HIV positive controls without invasive pneumococcal disease [7,8]. However, Amdahl and colleagues reported lower anti-pneumolysin titres in HIV positive individuals compared to HIV negative controls, opposite to the observation in our study. Sullivan and colleagues in their study of a cohort of intravenous drug users in the United States, found no significant difference in pre-disease anti-pneumolysin titres between cases and HIV negative control subjects [8]. Our study does differ from Amdahl's and Sullivan's as our cases were recruited after the individual had had an episode of pneumococcal bacteraemia, and followed up in time during which they suffered a recurrence; baseline serum anti-pneumolysin titres were obtained in the interval between two pneumococcal bacteraemia episodes. Therefore, as our cases had already suffered a pneumococcal event, it might be expected that they would have higher anti-pneumolysin titres.

A recent study of HIV infected African adults reported increased colonisation rates with *S. pneumoniae* [9]. Subjects colonized with *S.pneumoniae* have also been observed to have higher serum IgG concentrations to pneumolysin compared to patients with bacteraemic pneumococcal bacteraemia [6]. We therefore speculate that the increased rate of pneumococcal colonisation seen in HIV positive adults is a likely explanation for the observed higher anti-pneumolysin titres in HIV infected subjects in our study. We, however, did not investigate nasopharyngeal pneumococcal carriage in this study.

We expected an increase in pneumolysin titres following an episode of pneumococcal bacteraemia, as demonstrated in previous studies [6,10]. The change in anti-pneumolysin titres after pneumococcus re-infection in our study was not significantly different compared to baseline values. A weakness in our study could be the varying time intervals of sera collection before and after the recurrent infection. However, the median time interval of sera collection after the recurrent infection episode was 39 days; we think it unlikely that the expected rise in anti-pneumolysin titre could have been missed because of the timing of sera collection. It could therefore be suggested that HIV infected individuals show impaired response in anti-pneumolysin titres following pneumococcal bacteraemia. This may be a feature of the immune dysregulation in HIV infection, which is still not clearly understood.

Mucosal defence against pneumococcal carriage is dependent on the migration of CD4+ cells to the pulmonary mucosa in response to pneumolysin [11]. CD4− T-cell-negative mice are significantly more susceptible to pneumococcal lung infection and bacteraemia than mice with functional CD4+ T-cells [2]. Furthermore, low CD4+ T-cell levels have been shown to be associated with increased risk of bacteraemia in HIV infected patients with pneumococcal pneumonia [12]. In our study, the cases and HIV positive control groups had similar CD4+ cell counts (geometric mean < 200/μl) and therefore the influence of CD4+ cell level on susceptibility cannot be tested.

HIV infected individuals have higher specific IgG to pneumolysin in serum, in response to natural infection, compared to HIV uninfected subjects. However, invasive pneumococcal disease is common in HIV infected subjects despite this observed higher serum anti-pneumolysin titres. This observation of a quantitative immune response to pneumolysin may offer support to a pneumolysoid-based vaccine for HIV infected adults. However, these data suggest that pneumolysin antibody alone will not protect against naturally acquired invasive pneumococcal infection in this group. The role of pneumolysin may therefore be as an adjuvant or protein carrier of other vaccine antigens.

## Conflict of interests

None.

## Figures and Tables

**Table 1:** Demographics, CD4+ cell count, and anti-pneumolysin antibody titres in HIV infected cases with recurrent pneumococcal bacteraemia and control subjects.

	Cases (*n* = 34)	Group		Multiple comparison *p*-values^a^	
		HIV positive controls (*n* = 34)	HIV negative controls (*n* = 34)	Cases vs. HIV positive controls	Cases vs. HIV negative controls
Age (years), mean (s.d.)	35.4 (7.5)	35.0 (8.5)	32.9 (11.4.)	0.987	0.536
Sex *n* (Males/Females)	17/17	17/17	17/17		
CD4+ cell count/μl: geometric mean (95% CI)	132.9 (107.5–164.3)	178.2 (124.8–254.5)	568.3 (445.4–725.0)	0.286	<0.001
Pre-event anti-pneumolysin IgG titre (U/ml): geometric mean (95% CI)	849.1 (635.8–1134.0)	564.6 (428.6–743.8)	356.1 (296.3–428.0)	0.059	<0.001
Post-event anti-pneumolysin IgG titre (U/ml): geometric mean (95% CI)	840.2^b^ (610.9–1155.5)	–	–	–	–
Mean ratio of pre-event anti-pneumolysin IgG titre to post-event anti- pneumolysin IgG titre (95% CI)	1.007^b^ (0.818–1.240)^b^	–	–	–	–

a Tukey HSD *p*-values, adjusted for multiple comparisons.

b *n* = 29.
